# Detection of asymptomatic group A *Streptococcus* throat carriage and respiratory viruses during pharyngitis outbreaks in two daycare centers

**DOI:** 10.1128/spectrum.03802-25

**Published:** 2026-05-27

**Authors:** Kirsi Gröndahl-Yli-Hannuksela, Matti Waris, Riikka Österback, Jutta Peltoniemi, Mirva Virolainen, Kari Auranen, Teemu Kallonen, Kaisu Rantakokko-Jalava, Jaana Vuopio, Ville Peltola, Lauri Ivaska

**Affiliations:** 1 Institute of Biomedicine, University of Turku 8058, Turku, Finland; 2 Clinical Microbiology, Turku University Hospital 1041, Turku, Finland; 3 Wellbeing Services County of Southwest Finland 650546 , Turku, Finland; 4 Department of Mathematics and Statistics, University of Turku 8058, Turku, Finland; 5 Department of Clinical Medicine, University of Turku 8058, Turku, Finland; 6 Department of Paediatrics and Adolescent Medicine, Turku University Hospital and University of Turku 1041, Turku, Finland; 7 InFLAMES Research Flagship Center, University of Turku 8058, Turku, Finland; Michigan State University, E. Lansing, Michigan, USA

**Keywords:** pharyngitis, group A *Streptococcus*, enterovirus, outbreak, naat diagnostics, transmission

## Abstract

This study aimed to characterize the frequency of asymptomatic group A *Streptococcus* (GAS) carriage and respiratory virus detection, and to assess the utility of GAS nucleic acid amplification tests (NAATs) as a microbiological screening method during pharyngitis outbreaks. In this observational cross-sectional study, we recruited healthy children and daycare employees from two daycare centers experiencing GAS pharyngitis outbreaks. One throat swab was collected for GAS culture and NAAT, and another for respiratory virus NAAT. All GAS isolates underwent whole-genome sequencing (WGS). A total of 87 individuals were enrolled: 68 children and 19 adults. GAS was detected in 19/87 (22%) participants by culture, and 36/87 (41%) by NAAT. Most isolates were emm1.0 in outbreak 1 (5/7, 71%) and emm4.0 in outbreak 2 (10/12, 83%). WGS demonstrated high genetic similarity within each outbreak (median 1.3–3 SNP differences). Respiratory virus detection rates were similar between GAS-positive (10/19, 53%) and GAS-negative (33/68, 49%) individuals. In outbreak 1, enteroviruses were more common among GAS-positive participants (4/7, 57%) than GAS-negative participants (4/37, 11%; unadjusted *P* = 0.014). In conclusion, substantial asymptomatic GAS carriage was observed. The contribution of concurrent respiratory virus circulation to GAS transmission remains uncertain. However, enterovirus detection was more frequent among GAS-positive individuals in one outbreak. The high sensitivity of GAS NAATs, which detect nucleic acids rather than viable bacteria, may complicate efforts to balance outbreak control with antimicrobial stewardship.

**IMPORTANCE:**

Group A Streptococcus (GAS) is an increasingly important respiratory pathogen, causing illnesses ranging from life-threatening infections to pharyngitis and asymptomatic carriage. The factors influencing GAS transmission remain poorly defined, and, in the absence of a GAS vaccine, identifying determinants that may guide outbreak-control strategies is essential. In this observational cross-sectional study, substantial asymptomatic carriage was observed among children and adults in daycare centers experiencing GAS outbreaks: 22% by throat culture and 41% by nucleic acid amplification testing (NAAT). Co-detection of respiratory viruses was also common, and in one outbreak, enterovirus detection was more frequent among GAS-positive individuals. These findings provide preliminary insight into the complex dynamics of GAS transmission and highlight asymptomatic carriage and viral co-infections as potential intervention targets. However, the increased sensitivity of GAS NAATs compared with culture may complicate outbreak management by potentially driving unnecessary antimicrobial use.

## INTRODUCTION

*Streptococcus pyogenes* (group A *Streptococcus*, GAS) is an important bacterial cause of pharyngitis, impetigo, and invasive infections (1–3). As part of its phenotypic spectrum, asymptomatic throat carriage is well established and has been associated with impetigo cases within and between households in communities with a high burden of GAS infections (4–7). Prevalence of asymptomatic GAS throat carriage in children in high-income countries was estimated to be 10.5% in a recent meta-analysis (5). GAS can also cause outbreaks of pharyngitis or scarlet fever among children (8–10), although respiratory viruses are a more common cause of acute pharyngitis in this age group (11). Pharyngitis outbreaks in schools and daycare centers are often characterized by unclear microbiological etiology, repeated antibiotic prescriptions, and extensive use of diagnostic tests, placing a considerable burden on public health systems and clinical microbiology laboratories.

Viral co-infections play an important role in the pathogenesis and transmission of other bacterial respiratory pathogens, such as *Streptococcus pneumoniae* (12–16). However, the role of respiratory viruses in GAS pharyngitis, particularly in relation to diagnostics, transmission dynamics, and pathogenesis, remains less well understood and has only recently become a focus of research (17–19).

The aim of this study was to document the frequency of asymptomatic GAS carriage and the presence of respiratory viruses in the throat during suspected GAS pharyngitis outbreaks in daycare settings. Additionally, we evaluated the utility of GAS nucleic acid amplification tests (NAATs) as a diagnostic tool during outbreak investigations.

## RESULTS

### Outbreak description

Two suspected daycare center outbreaks were identified and included in this observational cross-sectional study during the surveillance period. Investigation for outbreak 1 was launched, and sampling took place in early April 2019, after several months of unusually frequent or repeated pharyngitis episodes reported in two daycare groups of the daycare center. For outbreak 2, investigation and sampling were launched in May 2019 after 2–3 weeks of increased pharyngitis incidence reported in four groups of the daycare center. Altogether, 37 children and seven adults in outbreak 1 and 31 children and 12 adults in outbreak 2 participated in the study (Table 1). None of the study participants had clinical pharyngitis during the sampling and were healthy enough to participate in daycare/work normally. Of all eligible children attending daycare during the outbreaks on the day of sampling, only one child in outbreak 2 did not consent to participate, resulting in an overall participation rate of 87/88 (99%) among eligible subjects. Following the public health intervention, which included throat culture screening and antibiotic treatment for GAS-positive individuals, neither daycare center reported ongoing pharyngitis outbreaks.

**TABLE 1:** Clinical characteristics of study participants in two group A *Streptococcus* (GAS) daycare outbreaks

	Outbreak 1 (*n* = 44)	Outbreak 2 (*n* = 43)	Total (*n* = 87)
Children, *n* (**%**)	37 (84)	31 (72)	68 (78)
Median age, year [IQR]	4.4 [3.8–5.2]	5.8 [4.1–6.2]	4.9 [4.0–5.8]
Sex (female), *n* (%)	19 (51)	10 (32)	29 (43)
Rhinitis or cough in previous 7 days, *n* (%)	17 (46)	12 (39)	29 (43)*^a^*
Antibiotic treatment in previous 14 days, *n* (%)	4 (11)	6 (19)	10 (15)
Household contact with confirmed GAS infection, *n* (%)	4 (11)	7 (23)	11 (16)
Adults, *n* (%)	7 (16)	12 (28)	19 (22)
Median age, year [IQR]	23.9 [22.2–30.7]	36.3 [31.7–44.2]	31.5 [26.7–40.1]
Sex (female), *n* (%)	7 (100)	12 (100)	19 (100)
Rhinitis or cough in previous 7 days, *n* (%)	6 (86)	5 (42)	11 (58)^*b*^
Antibiotic treatment in previous 14 days, *n* (%)	0	0	0
Household contact with confirmed GAS infection, *n* (%)	1 (14)	0	1 (5)

^*a*^ Rhinitis or cough on the day of sampling (*n* = 3).

^*b*^ Rhinitis or cough on the day of sampling (*n* = 0).

### GAS detection

**Fig 1:**
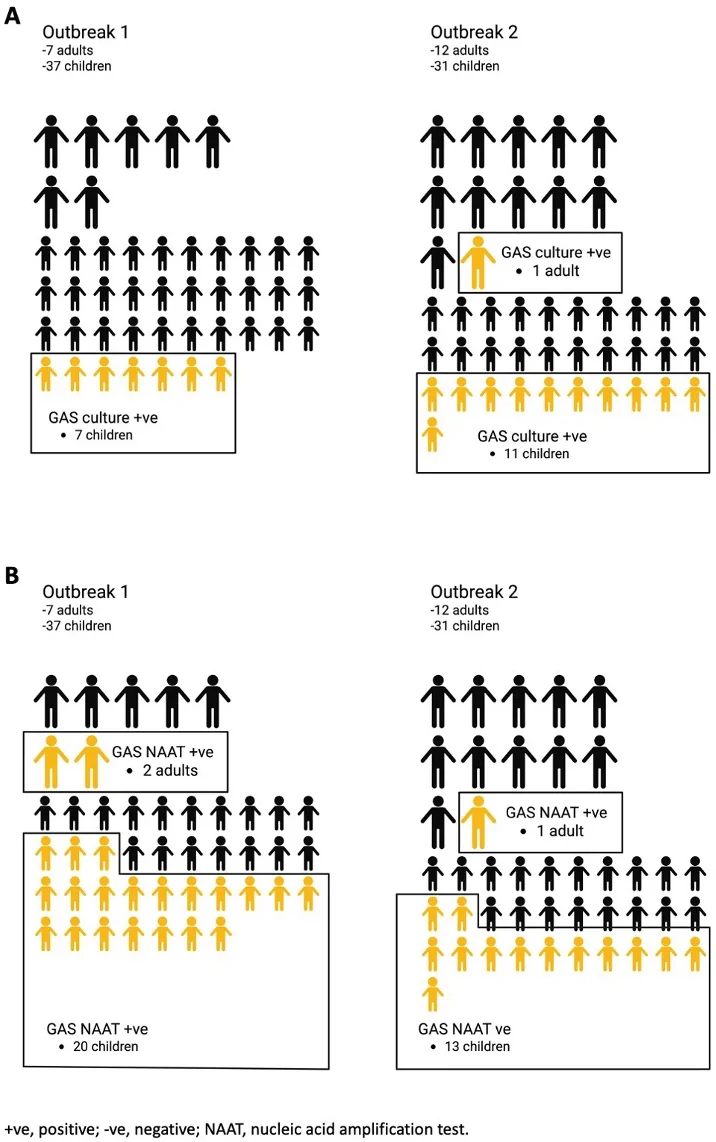
Schematic presentation of group A *Streptococcus* (GAS) findings based on throat culture (**A**) and nucleic acid amplification (**B**) during outbreaks in two daycare centers. Black figures represent GAS-negative and yellow figures GAS-positive individuals (created in BioRender).

Of the 44 swabbed subjects in outbreak 1, seven (16%) were culture-positive for GAS, and they were all children. In outbreak 2, 12 (28%) out of 43 subjects were GAS culture-positive, of whom one was an adult (Fig. 1). GAS NAAT testing identified 22 subjects (50%) as GAS-positive in outbreak 1 and 14 subjects (33%) in outbreak 2. All GAS culture-positive subjects were identified also with NAAT. There was a minor difference in the performance of the two NAATs used; GAS positivity was 41% and 34% with Solana and ID NOW Strep A2, respectively (Table S1). Most individuals with GAS detected by throat culture were asymptomatic: only 1/19 (5%) subjects with positive throat culture reported sore throat in the previous 7 days. In addition, 1/19 (5%) subjects with GAS-positive throat culture and NAAT, and one additional subject with solely GAS-positive NAAT, had cough on the day of sampling.

### Previous GAS exposure

A total of 10 subjects reported receiving antibiotic treatment within the previous 14 days: eight for pharyngitis or another GAS infection, and two for acute otitis media. None of these subjects tested GAS-positive by throat culture, and 2/10 were positive by NAAT. Additionally, 12 subjects (11 children and one adult) reported a prior GAS infection in a household member. Among them, three (25%) children were GAS-positive: three by NAAT, and two out of these three also by throat culture.

### GAS genotyping

All 19 GAS isolates were *emm* typed. In outbreak 1, *emm1.0* was predominant, identified in five of seven isolates (71%). In addition, one *emm1.25* and one *emm4.0* isolate were detected. In outbreak 2, 10 out of 12 isolates (83%) were *emm4.0*, and two were *emm28.0* (17%).

**Fig 2:**
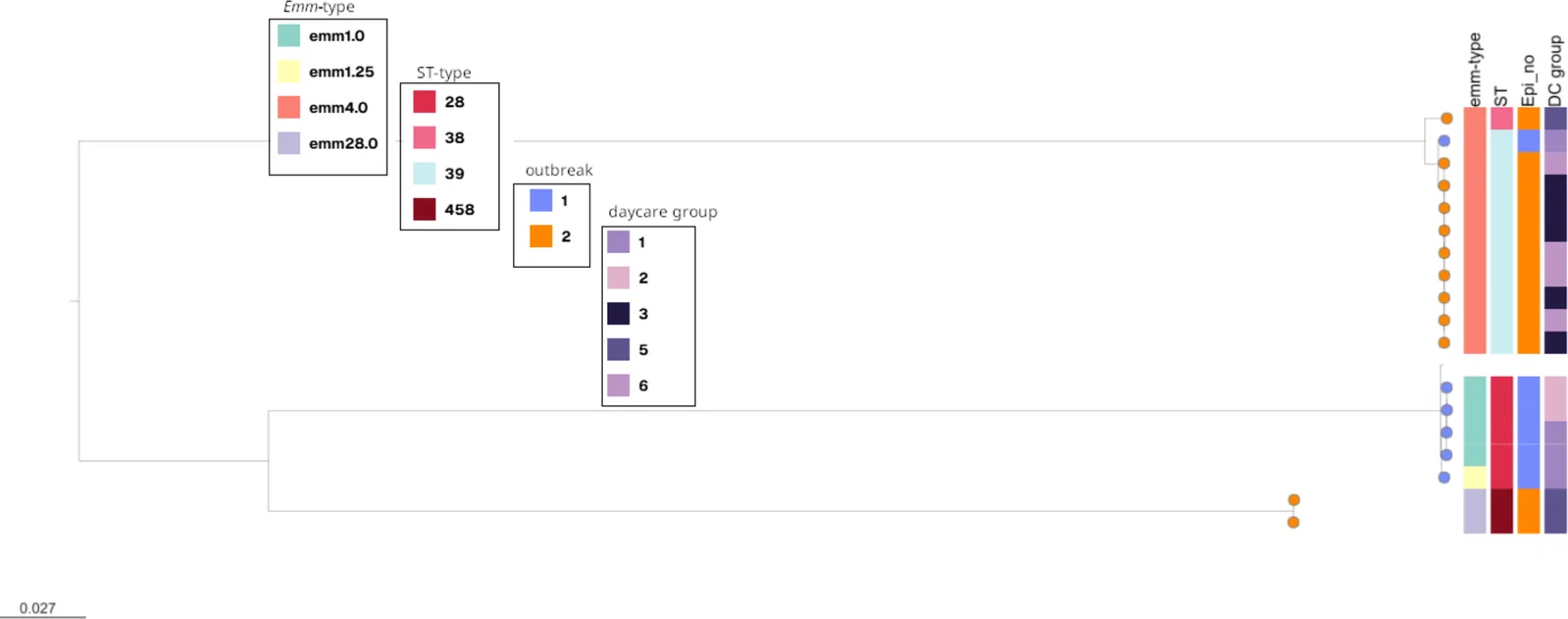
Phylogenetic relation of group A *Streptococcus* isolates identified from two outbreaks (*n*_total_ = 18, outbreak 1 in blue, outbreak 2 in orange). *emm*-types, sequence types (ST), and daycare group (DC groups) are shown.

Whole-genome sequencing was successfully performed on 18 isolates to assess strain clonality (Fig. 2). In outbreak 1, *emm1.0* isolates differed by a median of 1 SNP (range: 1–2 SNPs, Table S2). Similarly, *emm4.0* isolates from outbreak 2 were separated by a median of three SNPs. One *emm4.0* isolate in outbreak 2, which differed by sequence type (ST38) from the others (ST39), showed a larger genomic difference, with a median of 283 SNPs. A single *emm4.0* isolate from outbreak 1 differed from *emm4.0* isolates in outbreak 2 by a median of 73 SNPs. The two *emm28.0* isolates in outbreak 2 were closely related, with only a four-SNP difference.

Subjects from both outbreaks were distributed across six different daycare groups. GAS isolates from the same outbreak but different daycare groups were nearly identical, differing by only 1–4 SNPs, indicating strong clonality.

### Virus findings

In total, 24 subjects (55%) in outbreak 1 and 19 subjects (44%) in outbreak 2 had at least one virus detected in throat swabs (Table 2). Overall, virus detection was not significantly associated with GAS throat culture positivity. The most frequently identified respiratory viruses were rhinovirus, adenovirus, and enterovirus.

**TABLE 2:** Virus findings in group A *Streptococcus* throat culture-positive and -negative individuals

Throat culture result	Outbreak 1 (*n* = 44)		Outbreak 2 (*n* = 43)	
	GAS +ve (*n* = 7)	GAS −ve (*n* = 37)	GAS +ve (*n* = 12)	GAS −ve (*n* = 31)
Any respiratory virus positive, *n* (%)	5 (71)	19 (51)	5 (42)	14 (45)
Rhinovirus, *n* (%)	3 (43)	9 (24)	3 (25)	13 (42)
Adenovirus, *n* (%)	2 (29)	6 (14)	1 (8)	1 (3)
Enteroviruses, *n* (%)	4 (57)^a^	4 (11)^a^	0	0
Influenza A, *n* (%)	0	2 (5)	0	0
Bocavirus, *n* (%)	1 (14)	0	1 (8)	0
CoVOC43, *n* (%)	0	2 (5)	0	0
CoV229E, *n* (%)	0	1 (3)	1 (8)	0
CoVNL63, *n* (%)	0	1 (3)	0	0

^a^ Statistically significant difference (*P* < 0.05). GAS, group A *Streptococcus*; +ve, positive; −ve, negative; CoV, coronavirus.

**Fig 3:**
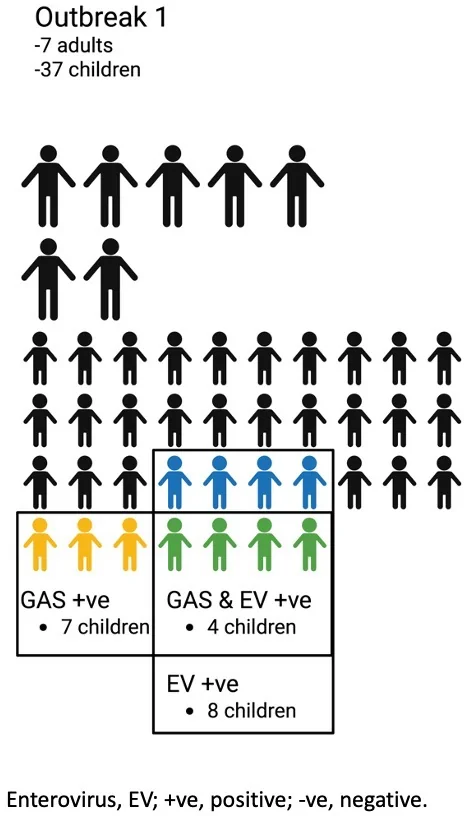
Cluster of enterovirus findings in group A *Streptococcus* (GAS) throat culture-positive children in outbreak 1. Black figures represent GAS- and enterovirus-negative (*n* = 33), yellow figures GAS-positive and enterovirus-negative (*n* = 3), blue figures GAS-negative and enterovirus-positive (*n* = 4), and green figures GAS- and enterovirus-positive (*n* = 4) individuals (created in BioRender).

In outbreak 1, enterovirus detection was associated with GAS positivity: enteroviruses were detected in four of seven GAS-positive subjects (57%), compared to four of 37 GAS-negative subjects (11%) (unadjusted *P* = 0.014; Table 2, Fig. 3). None of the enterovirus-positive subjects had respiratory symptoms at the time of sampling, but 3/8 (38%) reported rhinitis/cough in the preceding 7 days.

## DISCUSSION

In this observational study of two pharyngitis outbreaks in a daycare setting, asymptomatic GAS carriage and respiratory virus detection in the throat were prevalent among children. Overall, virus co-detection was not associated with asymptomatic GAS throat carriage. However, in the other outbreak, enteroviruses were detected more frequently in GAS-positive individuals than in GAS-negative individuals. Rapid NAATs were more sensitive than throat culture for GAS detection. If NAATs had been used as the microbiological screening method during the outbreak, a greater number of asymptomatic children would have been treated with antibiotics.

Previous studies have described a high proportion of asymptomatic GAS throat carriage during pharyngitis outbreaks in daycare and school settings, and our results reinforce these findings (8–10). Most of the asymptomatic GAS carriers identified in this study were children (Fig. 1), which emphasizes the relatively minor role of daycare employees in the transmission dynamics. In outbreak 1, where GAS transmission likely occurred over several weeks or months prior to intervention, lower bacterial densities or remnants of GAS could potentially explain the higher rate of GAS NAAT positivity (22/44, 50%) compared to throat culture (7/44, 16%). Another potential explanation for the difference in GAS detection by throat culture versus NAAT between the outbreaks could be related to distinct features of different dominant *emm* types (*emm1.0* vs *emm4.0*).

The detected *emm* types, primarily *emm1.0* and *emm4.0*, correlate with the temporal distribution of *emm* types observed in confirmed pharyngitis and invasive GAS cases in the same region of Finland (20). A recent study from Iceland reported the emergence of isolates with similar genetic backgrounds from both asymptomatic carriage and invasive infections, suggesting that, in children, carriage serves as a reservoir for both transmission and disease (21). In our study, whole-genome sequencing was used to confirm the clonality of the isolates within the outbreaks. The low SNP differences between isolates of the same *emm* type support the hypothesis of local GAS spread during the outbreak. However, single isolates with differing *emm* types within an outbreak could reflect true asymptomatic carriage unrelated to the ongoing outbreak.

The role of viral co-infection in the transmission of *Streptococcus pneumoniae* has been demonstrated in epidemiological models, controlled human infection studies, and observational cohort studies (14–16). Given prior evidence of viral facilitation of bacterial transmission in other respiratory pathogens, exploratory virus-specific analyses were undertaken to identify potential signals warranting confirmation. In this exploratory study, respiratory virus detection was not associated with simultaneous GAS throat carriage. Nevertheless, the detection of an enterovirus cluster in GAS-positive children during outbreak 1 suggests that respiratory viruses may increase the density of GAS throat carriage and thereby enhance its detection by throat culture, directly influence GAS transmission, or that GAS throat carriage increases the risk of enteroviral infection.

Screening all individuals with throat cultures and treating GAS-positive cases with 10 days of oral penicillin to control transmission remain controversial strategies (22–24). One aim of this study was to evaluate the utility of rapid GAS NAAT as a diagnostic alternative in screening healthy individuals in outbreak settings, where large volumes of concurrent throat samples are typically collected. Our findings show that the GAS detection rate was higher with NAATs than with throat culture, and that using NAAT for GAS screening might have resulted in more asymptomatic individuals receiving antibiotic treatment. It must be emphasized that these tests were designed to detect GAS in symptomatic individuals with acute pharyngitis and that, in this study, tests were evaluated outside their intended use. The outbreaks were contained with current practices; therefore, expansion of criteria for antibiotic treatment seems unnecessary. The higher analytical sensitivity of GAS NAATs compared to throat culture is consistent with previous findings in patients with acute pharyngitis (25, 26).

### Limitations and strengths

This study has several limitations. First, the small sample size, influenced by non-pharmaceutical interventions during the COVID-19 pandemic and the resultant absence of GAS circulation, limits the statistical power and generalizability of our findings. Second, we lacked a contemporaneous non-outbreak comparator, limiting our ability to quantify how much carriage exceeded baseline. Third, the cross-sectional design of the study precluded an evaluation of the impact of test-and-treat intervention on GAS throat carriage over time. Fourth, the commercial GAS NAATs used in this study provide only qualitative (positive vs negative) results without cycle threshold values, thus preventing evaluation of bacterial density. Finally, as the data were collected exclusively from healthy individuals, the results may not be directly applicable to settings where symptomatic pharyngitis is more common. Despite these limitations, the study has several notable strengths. The prospective design conducted during an active outbreak, combined with pharyngeal sampling for both respiratory virus diagnostics and GAS strain sequencing, provides a unique and comprehensive data set. These features enhance the robustness of our findings, offering insights into GAS carriage and viral co-infection in an outbreak setting.

### Conclusions

Our study highlights the significant burden of asymptomatic GAS throat carriage, which may serve as a reservoir for child-to-child transmission, repeated infections, and the high morbidity associated with pharyngitis outbreaks in daycare settings. It remains to be elucidated if respiratory virus co-infections facilitate GAS transmission and potentially accelerate the spread of the infection within the daycare environment. GAS NAATs detect bacterial nucleic acids instead of live bacteria, which may not be optimal for balancing outbreak containment with the judicious use of antibiotics. Our findings emphasize the need for prospective, longitudinal studies to further investigate the role of pharyngeal respiratory virus co-infections in shaping the clinical phenotype, transmission dynamics, and pathogenesis of GAS in both children and adults.

## MATERIALS AND METHODS

### Surveillance of GAS outbreaks

As part of routine communicable disease surveillance, daycare centers in Turku, Finland, report any unusual infection activity to the local public health team. If clinical assessment suggests a potential ongoing GAS outbreak, such as an increase in cases of pharyngitis, impetigo, or perianal dermatitis, a survey is distributed to the guardians of children attending the affected daycare unit. The purpose of the survey is to determine whether children have tested positive for GAS. According to the guidelines of the Finnish Institute for Health and Welfare, a GAS outbreak in a daycare setting (children aged 1–6 years) is suspected when ≥20% of children in a group are reported to have tested positive for GAS within a two-week period. Once this threshold is met, throat culture screening of all children and staff in the affected daycare groups is recommended to identify asymptomatic carriers. Individuals who test positive for GAS are advised to complete a 10-day course of oral penicillin, and hygiene measures are implemented to reduce further transmission.

### Study design, participants, and outcomes

We did an observational cross-sectional study in daycare centers undergoing GAS outbreak investigations in collaboration with the local public health team in Turku, Finland. On-site throat sampling was carried out for all children and staff who were healthy enough to be present in the affected daycare unit on the day of the investigation. Children absent on the sampling day were not included; reasons for absence (e.g., acute illness, recent antibiotic treatment) were not systematically recorded. The inclusion criteria for the study were the same as those for the public health intervention: children and staff who were present in the affected unit during the investigation. Mild symptoms (e.g., cough, rhinorrhea during the past 7 days) that did not prevent children from attending daycare on the day of sampling were not considered exclusion criteria. The study procedures included obtaining written informed consent from the guardians of eligible children and from adult daycare staff, collecting an additional throat swab for viral diagnostics alongside the routine throat culture, and administering a structured questionnaire to all participants.

Participants were recruited from suspected GAS outbreaks in daycare centers during the surveillance period between April 2019 and March 2021. However, it is important to note that the circulation of GAS and the incidence of pharyngitis outbreaks were substantially affected by non-pharmaceutical interventions implemented in response to the COVID-19 pandemic in Finland, starting in March 2020.

### Microbiological analysis

Throat swabs (ESwab, Copan Diagnostics Inc., Italy) were cultured on selective streptococcal agar, i.e., sheep blood agar (Oxoid/Thermo Fisher Scientific, USA) supplemented with colistin and oxolinic acid, for the identification of beta-hemolytic colonies. Suspected GAS isolates were initially confirmed using the Lancefield antigen agglutination test, and *S. pyogenes* was confirmed using a MALDI-TOF mass spectrometry instrument (Bruker Daltonics GmbH, Germany). GAS isolates identified, along with the original throat swabs, were transferred to the University of Turku for further *emm* typing and NAAT testing. All GAS isolates were *emm* typed following the protocols of the U.S. Centers for Disease Control and Prevention (CDC) (27). Isolates were processed using study codes and contained no personal identifiers.

Direct identification of GAS from throat swabs was performed using two commercial nucleic acid amplification tests: the Solana GAS assay (QuidelOrtho, USA) and the ID NOW Strep A2 assay (Abbott, USA). The tests were performed from the same swab used for bacterial culture, according to the manufacturers’ instructions as previously described (28).

Throat swabs (FLOQSwabs, Copan Diagnostics Inc., Italy) were collected and transported in dry sterile test tubes for virus detection. To extract total nucleic acids, each swab was swirled in 1 mL PBS, and 550 µL of the suspension was processed using NucliSENS easyMAG extractor (BioMerieux, The Netherlands) with a 55 µL elution volume. Viruses were detected using Allplex Respiratory Panels 1–3 (Seegene, South Korea), according to manufacturers’ instructions.

### Genomic analysis

For whole-genome sequencing, genomic DNA was extracted using NucleoSpin Microbial DNA Mini Kit (Macherey-Nagel, Germany). The sequencing was performed on an Illumina Nextseq2000 sequencer with 150 bp paired-end reads (Illumina, USA). The sequencing library was prepared with Nextera XT library preparation kit (Illumina, USA). The genomic analyses were performed on CLC Genomics Workbench (25.0) with the Microbial Genomics Module (QIAGEN Digital Insights, Denmark). The multi-locus sequence typing (MLST) analysis and mapping of reads against a reference genome (NCTC13751) were performed using “Type a Known species” pipeline. A maximum-likelihood phylogenetic tree and a SNP matrix were calculated using the “Create SNP Tree” pipeline with the GTR as the nucleotide substitution model, and the gamma distribution parameter was estimated.

### Statistical analysis

Descriptive statistics are presented as percentages. For comparisons of categorical variables, the Chi-square test with Yates’ continuity correction or Fisher’s exact test was applied, as appropriate. A *P*-value < 0.05 was considered statistically significant. All statistical analyses were performed using RStudio (version 2024.12.0 + 467; R Foundation for Statistical Computing, Vienna, Austria) with the “stats” package.

## Data Availability

All sequencing data are deposited into the National Center for Biotechnology Information Sequence Read Archive under BioProject PRJNA1458093.

## SUPPLEMENTAL MATERIAL

Tables S1 and S2 spectrum.03802-25-s0001.pdf Table S1: Diagnostic performance of GAS NAATs in comparison with throat culture. Table S2: SNP matrix of GAS isolates included in WGS analysis.

ASM does not own the copyrights to Supplemental Material that may be linked to, or accessed through, an article. The authors have granted ASM a non-exclusive, world-wide license to publish the Supplemental Material files. Please contact the corresponding author directly for reuse.
