# Supplementary material for: Detection of asymptomatic group A Streptococcus throat carriage and respiratory viruses during pharyngitis outbreaks in two daycare centers
Source: Microbiol Spectr. 2026 May 27;14(7):e03802-25. doi: 10.1128/spectrum.03802-25 (PMC13339969; doi:10.1128/spectrum.03802-25)
Supplement: Tables S1 and S2 — Table S1: Diagnostic performance of GAS NAATs in comparison with throat culture. Table S2: SNP matrix of GAS isolates included in WGS analysis. [file spectrum.03802-25-s0001.pdf]

Online Supplement to:

Detection of asymptomatic group A *Streptococcus* throat carriage and respiratory viruses during pharyngitis outbreaks in two daycare centers

Kirsi Gröndahl-Yli-Hannuksela, Matti Waris, Riikka Österback, Jutta Peltoniemi, Mirva Virolainen, Kari Auranen, Teemu Kallonen, Kaisu Rantakokko-Jalava, Jaana Vuopio, Ville Peltola, Lauri Ivaska

Supplementary table 1. Diagnostic performance of two different group A *Streptococcus* point-of-care nucleic acid amplification tests in comparison with throat culture.

Supplementary table 2. Single nucleotide polymorphism matrix of group A *Streptococcus* isolates included in the whole genome sequencing analysis.

Supplementary table 1. Diagnostic performance of two different group A *Streptococcus* (GAS) point-of-care nucleic acid amplification tests (NAATs) in comparison with throat culture (n=87).

		**Performance, % (95% CI)**				
	**GAS positive samples, n (%)**	**Sensitivity**	**Specificity**	**PPV**	**NPV**	**Accuracy[a]**
**Throat culture**	19 (22)					
**NAAT tests**						
Solana	36 (41)	100	75 (66-84)	53 (42-63)	100	80 (72-89)
Alere i	30 (34)	100	84 (76-92)	63 (53-73)	100	87 (80-94)

CI, confidence interval; PPV, positive predictive value; NPV, negative predictive value; NAAT, nucleic acid amplification technique

[a] Accuracy = the percentage of correctly classified samples

Supplementary table 2. Single nucleotide polymorphism (SNP) matrix of group A *Streptococcus* isolates included in the whole genome sequencing analysis (n=18). SNP difference between isolates <100 (pale orange), SNP difference 100-500 (light magenta), SNP difference >500 (dark magenta).

			**outbreak1**						**outbreak2**											
	Sample_id		1	2	3	4	5	6	7	8	9	10	11	12	13	14	15	16	17	18
		emm-type	emm1.0	emm1.0	emm4.0	emm1.25	emm1.0	emm1.0	emm4.0	emm4.0	emm28.0	emm4.0	emm4.0	emm4.0	emm28.0	emm4.0	emm4.0	emm4.0	emm4.0	emm4.0
**outbreak1**	1	emm1.0	0	1	10933	60	2	1	10937	10924	9777	10923	10924	10923	9777	10924	10924	10924	10925	10923
	2	emm1.0	1	0	10934	59	1	0	10938	10925	9778	10924	10925	10924	9778	10925	10925	10925	10926	10924
	3	emm4.0	10933	10934	0	10929	10935	10934	293	73	10630	70	72	71	10632	74	73	71	73	73
	4	emm1.25	60	59	10929	0	60	59	10933	10920	9775	10919	10920	10919	9775	10920	10920	10920	10921	10919
	5	emm1.0	2	1	10935	60	0	1	10939	10926	9779	10925	10926	10925	9779	10926	10926	10926	10927	10925
	6	emm1.0	1	0	10934	59	1	0	10938	10925	9778	10924	10925	10924	9778	10925	10925	10925	10926	10924
**outbreak2**	7	emm4.0	10937	10938	293	10933	10939	10938	0	282	10633	283	285	282	10635	283	284	282	282	284
	8	emm4.0	10924	10925	73	10920	10926	10925	282	0	10621	5	3	2	10623	3	4	2	2	4
	9	emm28.0	9777	9778	10630	9775	9779	9778	10633	10621	0	10620	10621	10620	4	10621	10621	10621	10622	10620
	10	emm4.0	10923	10924	70	10919	10925	10924	283	5	10620	0	4	5	10622	4	3	5	5	3
	11	emm4.0	10924	10925	72	10920	10926	10925	285	3	10621	4	0	3	10623	6	5	3	5	5
	12	emm4.0	10923	10924	71	10919	10925	10924	282	2	10620	5	3	0	10622	5	6	2	4	6
	13	emm28.0	9777	9778	10632	9775	9779	9778	10635	10623	4	10622	10623	10622	0	10623	10623	10623	10624	10622
	14	emm4.0	10924	10925	74	10920	10926	10925	283	3	10621	4	6	5	10623	0	3	5	1	3
	15	emm4.0	10924	10925	73	10920	10926	10925	284	4	10621	3	5	6	10623	3	0	4	4	2
	16	emm4.0	10924	10925	71	10920	10926	10925	282	2	10621	5	3	2	10623	5	4	0	4	4
	17	emm4.0	10925	10926	73	10921	10927	10926	282	2	10622	5	5	4	10624	1	4	4	0	4
	18	emm4.0	10923	10924	73	10919	10925	10924	284	4	10620	3	5	6	10622	3	2	4	4	0

SNP difference
<100
100-500
>500
